# A Graph-Based Framework for Multiscale Modeling of Physiological Transport

**DOI:** 10.3389/fnetp.2021.802881

**Published:** 2022-01-12

**Authors:** M. Deepa Maheshvare, Soumyendu Raha, Debnath Pal

**Affiliations:** Department of Computational and Data Sciences, Indian Institute of Science, Bangalore, India

**Keywords:** biophysical interactions, discrete network model, functional networks, hierarchical modeling, microvasculature and microenvironment, solute transport, spatio-temporal dynamics, systems biology

## Abstract

Trillions of chemical reactions occur in the human body every second, where the generated products are not only consumed locally but also transported to various locations in a systematic manner to sustain homeostasis. Current solutions to model these biological phenomena are restricted in computability and scalability due to the use of continuum approaches in which it is practically impossible to encapsulate the complexity of the physiological processes occurring at diverse scales. Here, we present a discrete modeling framework defined on an interacting graph that offers the flexibility to model multiscale systems by translating the physical space into a metamodel. We discretize the graph-based metamodel into functional units composed of well-mixed volumes with vascular and cellular subdomains; the operators defined over these volumes define the transport dynamics. We predict glucose drift governed by advective–dispersive transport in the vascular subdomains of an islet vasculature and cross-validate the flow and concentration fields with finite-element–based COMSOL simulations. Vascular and cellular subdomains are coupled to model the nutrient exchange occurring in response to the gradient arising out of reaction and perfusion dynamics. The application of our framework for modeling biologically relevant test systems shows how our approach can assimilate both multi-omics data from *in vitro*–*in vivo* studies and vascular topology from imaging studies for examining the structure–function relationship of complex vasculatures. The framework can advance simulation of whole-body networks at user-defined levels and is expected to find major use in personalized medicine and drug discovery.

**GRAPHICAL ABSTRACT:**
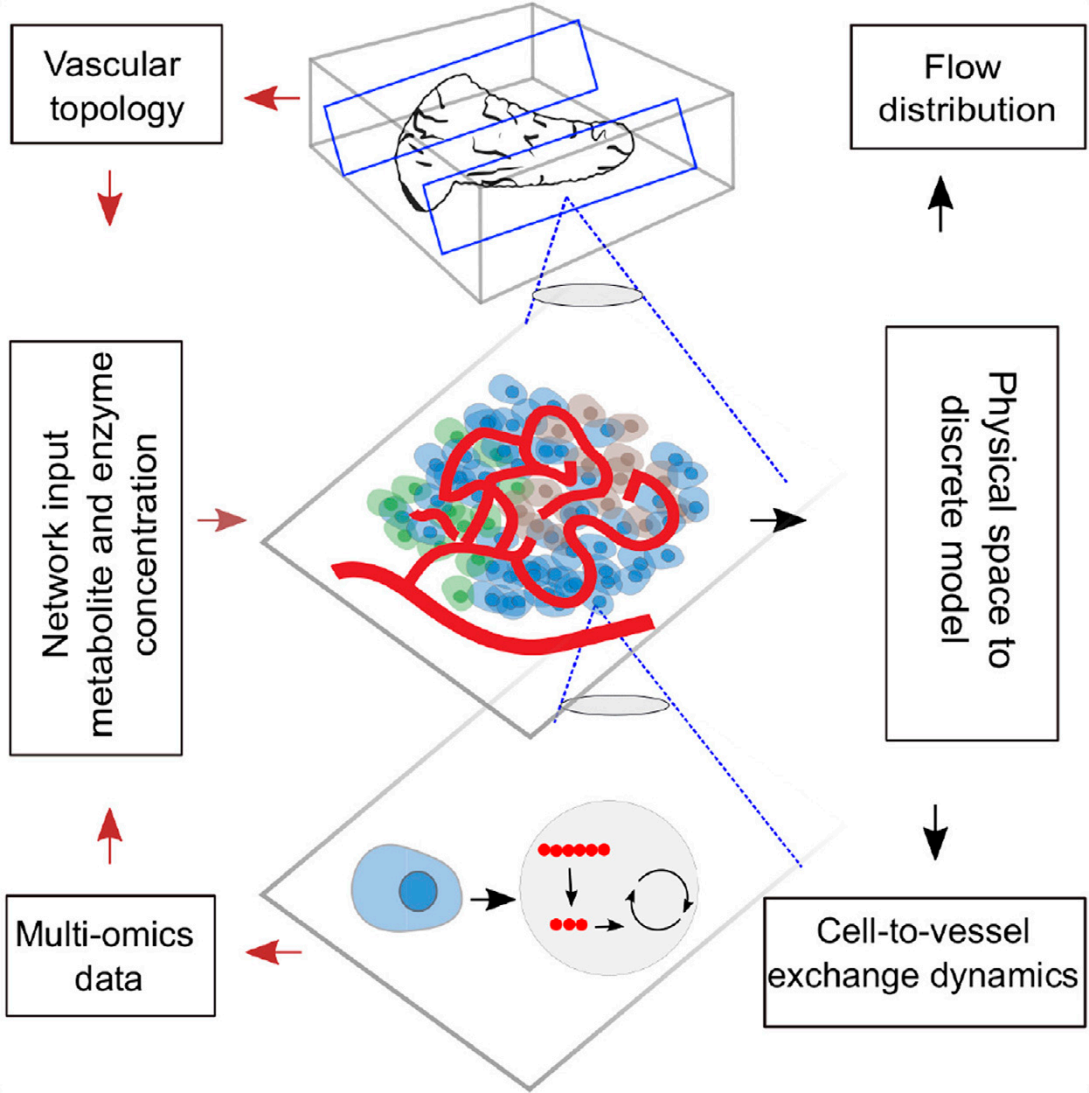


## 1 Introduction

The physiological system is a complex network in which each organ forms a subsystem and different subsystems interact to maintain overall homeostasis of the body. Within each subsystem, functional networks exist at different levels of complexity. Metabolic and signaling networks within a cell, cell-to-cell communication networks in the extravascular region of tissue, cell-to-vessel communication networks, and the vascular network which couples the local dynamics to the global dynamics determine the functional behavior of all tissues. Bottom-up and top-down modeling approaches emulate the cellular dynamics and organ-level physiology. The ability to simultaneously capture the local and global dynamics by hierarchically bridging the communication networks existing across diverse scales is the key challenge in the holistic modeling of physiology.

Microscale cellular models use a bottom-up approach in which multi-omics data assimilated from high-throughput sources are employed to formulate and validate parameter-intensive kinetic models. These models capture the dependency of intracellular dynamics on metabolic steady states and flux distributions (König et al., 2012; Berndt et al., 2018a; Masid et al., 2020). Owing to cellular heterogeneity and the existence of intercellular communication, autocrine and paracrine signaling (Koh et al., 2012; Watts et al., 2016; Rao and Rizzo, 2020), the response elicited by a single cell, cannot be scaled to a cell community. Cell population models, therefore, employ discrete modeling approaches for examining cell-to-cell interactions such as intra- and inter-islet synchronization established by gap junctional coupling (Pedersen et al., 2005; Barua and Goel, 2016).

Macroscopic organ scale compartment models (Sorensen, 1985) employ a top-down modeling approach for predicting the bulk flow and elimination kinetics of biomolecules. These organ scale models rely on single-tube, parallel-tube, or tank-in-series approximations for idealizing distribution volume of blood into compartments (Gray and Tam, 1987). For improving the mechanistic understanding of tissue–vessel interaction, multiphase porous media–based models representing the tissue volume as intravascular and multi-region extravascular compartments (e.g., capillary–interstitial–parenchymal exchange unit) (Deussen and Bassingthwaighte, 1996; Chalhoub et al., 2007) emerged. However, these frameworks do not offer the possibility to fuse macroscale and microscale models. Consequently, the effect of network architecture on microperfusion patterns (Dolenšek et al., 2015) and its influence on the nutrient exchange cannot be investigated by these compartment models.

To overcome the limitations of compartment-based models, continuum approaches have been put forth for understanding the implications of morphological changes on the functional response of an organ. In the extravascular domain, continuum approaches are helpful in estimating the collective response of a tissue mass where the bulk of the tissue is smeared and treated as a homogenous domain. Although homogenization simplifies the complexity of the computational domain, the approach is limited in its ability to probe aspects such as the influence of heterogenous arrangement of cells on nutrient release patterns. In the intravascular domain, continuum approaches are suitable for analyzing the effects of dilation of blood vessels, deformations that occur as a result of fluid–structure interactions. With the advancements in imaging studies, the availability of microvascular datasets offers the possibility to model large-scale networks. However, discretizing the tortuous microvasculature vasculature for 3D modeling of the advection–dispersion physics gives rise to extensive computational overhead while employing continuum approaches. This has led to the development of graph- and hybrid-based approaches in which the vasculature infiltrating the tissue volume is represented as a one-dimensional network of pipes for modeling the flow and delivering resources over networks (Beard and Bassingthwaighte, 2000; Fang et al., 2008; Heaton et al., 2012; Kojic et al., 2017; Safaei et al., 2018; Erlich et al., 2019). In summary, while efforts have been made to resolve: 1) spatial heterogeneity at subcellular scale (Ii et al., 2019), 2) short-range communication in the microenvironment of cell communities, and 3) metabolic zonation in single sinusoid models (Berndt et al., 2018b), explicit models of long-range communications mediated by the vascular system remain underdeveloped at both intra-organ and inter-organ scale.

Toward this end, we need a scalable hierarchical framework that allows us to bridge diverse scales for modeling production, consumption, and distribution of biochemicals in a tissue microenvironment. We introduce a discrete modeling framework for simulating gradient-driven advection–dispersion-reaction physics of multispecies transport. Graph-theoretic approaches that have been proven successful in examining flow of information through large-scale real-world networks are applied (Kumar et al., 2019; Bellocchi and Geroliminis, 2020; Besse and Faye, 2021) in this study. We resort to discrete-vector calculus and use the operators defined on a finite-graph to spatially discretize and formulate the transport dynamics in the vascular domain as a “tank-in-series” model. Further, the computational domain for establishing the vessel-to-cell exchange and cellular dynamics within the cell are set up by combining ideas from other multiscale and Krogh cylinder models (Berndt et al., 2018b; Frost et al., 2019). Dynamics of nutrient exchange from the blood vessel to the layer of cells that lie in close proximity to the vessel surface is modeled; cellular reactions are explicitly modeled by representing cells as discrete volume nodes. Differential equations defining the interactions over nodal volumes embedded in the graph are solved by translating the physical domain into a metamodel in which the biophysical attributes are subsumed. This framework is suitable for the following key applications: 1) to reduce the computational cost involved in the spatial discretization of large tissue volumes (Section 3.1.2); our discrete approach is geared toward obtaining fast solution by reducing the system dimension, and the metamodel is scalable into any domain; 2) to probe the effect of flow topology on scalar transport and the sensitivity of concentration dynamics to network parameters and variations in physiological set points. (Section 3.2, 3.4); and 3) to assimilate multi-omics data from *in vitro* and *in vivo* studies and vascular topology from imaging studies (Section 3.3) for examining the influence of structural changes on the functional response of a tissue. Our graph-based discrete modeling framework differs from the existing approaches in the following aspects: conventional finite-difference, finite volume– or finite-element–based formulations operate on a continuous domain, and the equations are discretized, and approximate solutions are obtained. In our approach, we discretize the physical space and solve the equations on the graph which forms the discrete domain. This makes it possible to scale our framework to large networks and offers the flexibility to fuse multiscale models by encoding imaging data of vascular topology and omics data of cellular reactions to enhance systems-level understanding.

The outline of this study is as follows: the procedure followed in translating the capillary vasculature into a weighted graph and the preliminary assumptions considered for setting up the computational domain are discussed in Sections 2.1 and 2.2. The governing equations of the flow distribution and the mathematical formulation of the discrete model of advection–diffusion-reaction physics are presented (Sections 2.3, 2.4, 2.5). We use our framework to model two physiologically relevant test systems: 1) advection–dispersion dynamics of glucose transport in the microvasculature and 2) advection–dispersion-reaction dynamics of glucose–lactate exchange in the functionally coupled tissue–vascular domains (glucose–lactate dynamics is relevant in tumor metabolism where metabolic activity alters in tumor microenvironments (Yang et al., 2021) and in modeling fuel-stimulated insulin secretion (Jiang et al., 2007; Prentki et al., 2013)). By applying our method, we predict glucose drift in the islet vasculature and cross-validate the flow and concentration fields of the multiphysics simulation with COMSOL simulations (Section 3.1.1). We establish the cell–vessel link and predict the spatio-temporal evolution of glucose–lactate exchange in the extravascular and intravascular domains (Section 3.3). We test the model behavior for various flow topologies (Section 3.2) and different pressure drops and glucose doses (Section 3.4). The network configurations illustrated in the applications presented in this work are the capillary blood vessels.

## 2 Methodology

**FIGURE 1:**
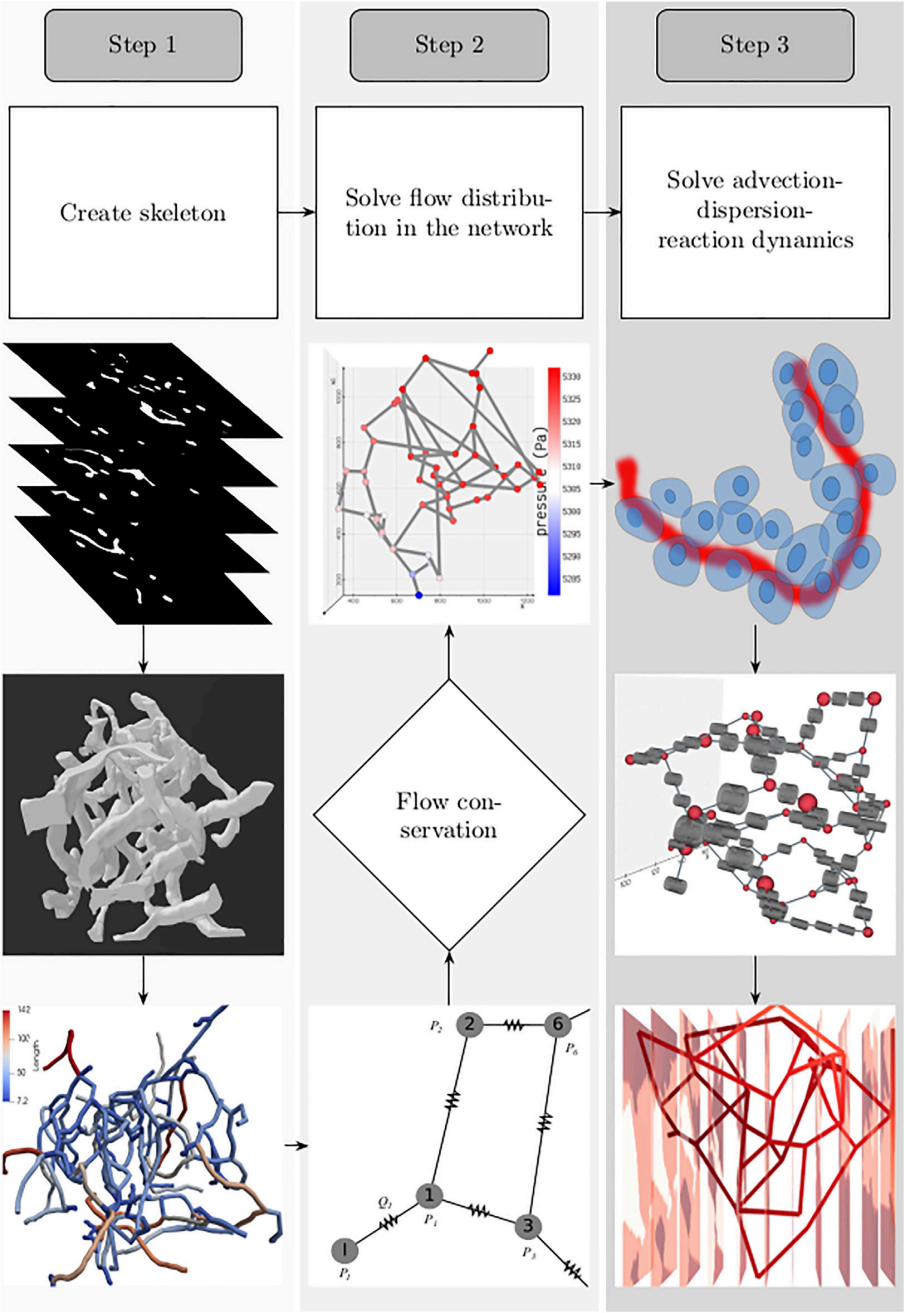
Workflow involved in setting up the system for simulating the spatio-temporal evolution of biochemical species is illustrated: *1. Create skeleton*: top, z-stack of microscopic images; middle, three-dimensional volume of the reconstructed vascular geometry; bottom, the length of blood vessel branches are color-coded. *2. Solve flow distribution in the network*: bottom, the vascular network represented as a hydraulic circuit; middle, from the estimated nodal pressures, flow through the vessel branches are computed, and checked whether the mass conservation holds at the junctions; top: 3D visualization of the pressure gradient observed across the vasculature. *3. Solve advection–dispersion-reaction dynamics*: top, creation of the computational domain which includes the blood vessel and the layer of the cells located in the vicinity of the outer surface of the capillary; middle, functional unit of the computational domain consisting of the vascular and cell sub-domains; bottom, concentration gradient observed across the vasculature after solving the advection–dispersion-reaction dynamics.

For setting up the discrete modeling framework to study the multiphysics coupling in multiscale systems, we start by introducing the steps involved in constructing the computational domain which is a metamodel of the physical space, dissection of the metamodel into subdomains which form the functional units, and formulation of the mathematical operators. The three main steps involved in our workflow are illustrated in Figure 1. 1) *Create skeleton*: The topological organization of the capillary network and biophysical characteristics such as length and diameter of the vessels in the network constitute the structural and anatomical characteristics relevant for setting up the computational domain. These characteristics are extracted in this step by skeletonizing the reconstructed vasculature (Section 2.1). 2) *Solve flow distribution in the network*: The physical space is translated into a weighted graph representing a hydraulic circuit. The pressure and flow fields are computed over the network by establishing the relationship between the node and edge entities of the graph using the Hagen–Poiseuille equation (Section 2.3). 3) *Solve advection–dispersion-reaction dynamics*: The metamodel is subdivided into functional units composed of cell and vessel subdomains. We combine multiple scales by coupling the uptake and release flux of the cell domain *ω* ^*t*^ with the carrier-mediated exchange flux occurring at cell–vessel interface. The metabolic reactions occurring at the cellular scale are modeled by biochemical rate laws, and the mass transport in the capillary domain Ω^*bv*^ is described by coupling the cell-to-vessel influx or outflux with the gradient-driven advection–dispersion transport in the capillary domain Ω^*bv*^ (Section 2.4, 2.5). Henceforth, the superscripts *bv* and *t* denote the parameters and variables defined in the capillary blood vessel and tissue domains, respectively.

### 2.1 Construction of Capillary Networks

**FIGURE 2:**
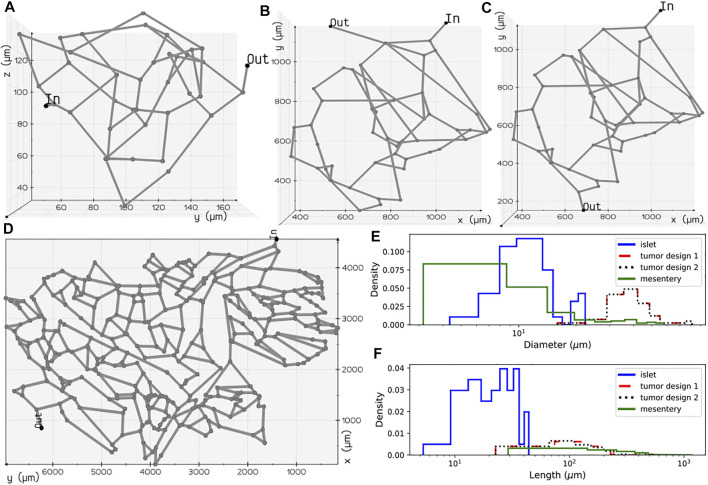
Microvascular network configurations: **(A)** pancreatic islet, **(B)** tumor design 1, **(C)** tumor design 2, and **(D)** mesentery generated after skeletonization of 3D volumes reconstructed from the image geometries studied in Chen et al. (2020) and d’Esposito et al. (2018). Diameter **(E)** and length **(F)** distributions of the vessel segments present in the four geometries.

The topology of the blood vessels that exchange nutrients with the surrounding tissue is represented by the one-dimensional network of pipes. The biophysical attributes, such as diameter and length of each segment and the blood vessel fragment connecting two branching points, are quantified as edge weights ( W ) of the graph network ( G ). We investigate four network configurations in this work. The 3D vasculature of pancreatic islet displayed in Figure 2A and both the configurations of tumor network shown in Figures 2B,C were reconstructed from binary images of vessels which were examined in Chen et al. (Chen et al., 2020). The tiff stacks containing binary images were generated in their study by imaging the blood vessels, labeled with a fluorescent dye, using light-sheet microscopy followed by segmentation of the vessels in the *ilastik* (Sommer et al., 2011) toolkit which leverages a machine learning–based classification algorithm. We follow the workflow detailed below for generating a weighted graph from the binary images: 1) the multipage .tiff image was rendered into a 3D volume using the ray-casting technique available in *3D Slicer* (Fedorov et al., 2012) by specifying the spacing of the image stack in the xyz directions. The dimensions of the input stack and domain size of the reconstructed volume are given in Table 1. 2) The largest connected region of the segmented volume was filtered using the *Island* effect available in *Slicer*, and the 3D object was exported in a stereolithography file (.stl) for skeletonization in the *Vascular Modeling Toolkit library (VMTK)* (Antiga et al., 2008). 3) *vmtksurfaceclipper* was employed to open the surface at the network inlet, and the *vmtknetworkextraction* algorithm was utilized to skeletonize the geometry. This yielded a network with nodes (vertices) ( V ) positioned at the N-furcation points or terminal ends and edges ( E ) formed by the vessel segment linking two nodes. 4) Segment length *l* ^*bv*^ and diameter *d* ^*bv*^ of the blood vessels were computed by tracing the shortest path between two nodes and extracting the maximum inscribed sphere radius (thickness), respectively. The 2D structure of the mesentery vasculature displayed in Figure 2D was generated by parsing the diameter, length of vessels, and topology information available in *Amira* mesh file provided in Esposito et al. (d’Esposito et al., 2018). Statistics of diameter and length distributions are shown in Figures 2E,F. We represent the skeletonized geometry of the capillaries as a weighted graph, $G\left(V\in R^{m},E\in R^{n},W\in R^{n}\right)$ , for investigating the advection–diffusion-reaction dynamics. The cardinality of all the properties and the operators defined on G are summarized in Supplementary Table S1.

**TABLE 1:** Specifications of the computational domain, values of flow boundary conditions, and the values of transport parameters used in the model.

Tissue (unit)	Islet	Tumor design 1 and 2	Mesentery
Image dimensions	—	—	—
x (pixels)	366	529	—
y (pixels)	366	529	—
z (pixels)	253	348	—
Image spacing (*μm*)	0.6	2.31	—
Domain size	—	—	—
x (*μm*)	219.6	1,221.9	—
y (*μm*)	219.6	1,221.9	—
z (*μm*)	151.8	803.8	—
# Vessel segments	52	63	489
*v*_*in*_ (*μm*/s)	160^*a*^	400^*b*^	400^*b*^
*Q*_*in*_/*Q*_*out*_ (nl/min)	3.76	23.8	40
*P*_*in*_ (mmHg)	60	60	-
*P*_*out*_ (mmHg)	—	—	0
*ν* (Pas)	0.004^*c*^	0.004^*c*^	0.004^*c*^
${\tilde{D}}_{A}$ (*cm* ^2^/min)	5.46e−4^*c*^	5.46e−4^*c*^	5.46e−4^*c*^
${\tilde{D}}_{B}$ (*cm* ^2^/min)	7.71e−4^*c*^	7.71e−4^*c*^	7.71e−4^*c*^

#—number; *v*_*in*_—inlet velocity; *Q*_*in*_—inflow rate; *Q*_*out*_—outflow rate; *P*_*in*_—inlet pressure; *P*_*out*_—outlet pressure; *ν*—viscosity of blood; ${\tilde{D}}_{A}$ —diffusion coefficient of species A (glucose) in blood; ${\tilde{D}}_{B}$ —diffusion coefficient of species B (lactate) in blood; a—Diez et al. (2017); b—Gabriel et al. (2020); c—Berndt et al. (2018b).

### 2.2 Preliminary Assumptions

**FIGURE 3:**
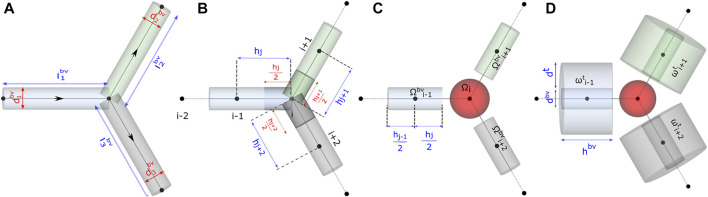
Schematic representation of functional units in the model. Computational domain for studying **(A)** flow distribution, **(B)** and **(C)** advective–dispersive transport of biochemicals in the discrete volumes of the blood vessel Ω^*bv*^, and **(D)** advective–dispersive-reactive transport of biochemicals in the discrete volumes of the blood vessel Ω^*bv*^ and tissue domains *ω* ^*t*^.

The time-dependent uptake and release of biochemicals by a tissue is determined by the gradient-driven transport across the capillary vessels which facilitate the transvascular exchange of solute with the tissue interstitium. Subsequently, the carrier-mediated sites present on the plasma membrane of cells aid the uptake of nutrient resources from the interstitial space for metabolism. Here, we introduce a graph-based mathematical framework for capturing blood-tissue exchange. The following simplifying assumptions are made in our model similar to the assumptions considered in the other multiscale studies: 1) assuming that the scalar concentration in the interstitial fluid attains rapid equilibrium with the concentration in the blood (Chalhoub et al., 2007), the interstitial compartment is not modeled; 2) the endothelial layer of the capillary surface is lined with metabolite transporters, which promote facilitated diffusion of biochemicals across the capillary wall; a similar approach has been presented in Heaton et al. (Heaton, 2012). We consider this as a reasonable assumption since a large fraction of the endocrine cells of the pancreas lie in close contact with the surface of the capillaries (Cohrs et al., 2017); 3) due to the deficiency of lymphatic drainage in the islets of Langerhans (Korsgren and Korsgren, 2016), fluid exchange with lymph vessels is not modeled (Thurber and Weissleder, 2011). Based on these considerations, we subdivide each blood vessel branch and the layer of tissue surrounding the capillary into discrete functional units, diagrammed in Figure 3D. The molar transport occurring through the blood vessel compartment of these functional units is modeled by the one-dimensional advection–dispersion equation (Taylor, 1954) that accounts for convection flux and the axial and radial diffusive flux of the solute molecules. The transcapillary exchange flux and the cellular processes occurring in the tissue compartment of each functional unit are modeled by rate expressions that capture the kinetics of metabolite-specific transporters and enzyme-catalyzed reactions, respectively. The governing equations that model the inter-compartment dynamics of these well-mixed volumes embedded in the finite connected network representation of the capillary bed is detailed in Sections 2.4 and 2.5.

### 2.3 Mathematical Formulation of Flow Distribution in the Network

To simulate the spatio-temporal evolution of chemical species, we first solve for the flow field in the vascular branches using the approach generally applied in studies that focus on modeling flow distribution in branching networks (Kirkegaard and Kim, 2020; Poelma, 2017; d’Esposito et al., 2018; Erlich et al., 2019).

#### 2.3.1 Domain

To perform fluid flow simulations, the oriented graph depicted in Figure 3A was used as the computational domain for setting up the linear system of equations. As illustrated, each blood vessel branch was treated as an axially symmetric cylinder of axial length *l* ^*bv*^ and circular radius *r* ^*bv*^ derived from the diameter *d* ^*bv*^ extracted from the skeletonized geometry (Section 2.1).

#### 2.3.2 Equations

We consider blood as an incompressible, viscous Newtonian fluid and apply the Hagen–Poiseuille equation (Eq. 2) for modeling the conductance of an edge as a function of viscosity *μ*, radius *r* ^*bv*^, and length *l* ^*bv*^. The linear analysis of the flow distribution presented here is applicable for laminar flow, *Re* < 1 in all segments, and this can be extended further to study the nonlinear rheology of blood using the procedure illustrated in Pries et al. (Pries et al., 1990). Therefore, the distribution of flow in a microvascular network is determined by the pressure gradient and the resistance offered to flow:

$$Q_{ij}=\frac{1}{R_{ij}}\left(p_{i}-p_{j}\right).$$ (1)

Here, *p*_*i*_ and *p*_*j*_ are the pressures at tail *i* and head *j* nodes of the oriented edge *e*_*ij*_, and *G*_*ij*_ is the conductance associated with *e*_*ij*_.

$$Q_{ij}=G_{ij}\left(p_{i}-p_{j}\right)=\frac{\pi {\left(r^{bv}\right)}^{4}}{8\;\mu l^{bv}}\Delta p.$$ (2)

The vector $Q\in R^{n}$ of volumetric flow rates in *n* edges is defined in Eq. 3. The negative of the oriented incidence matrix $M\left(G\right)\in R^{n\times m}$ , denoted as *M* henceforth, is the gradient operator that acts on the vector $P\in R^{m}$ of nodal pressures to result in the vector $\Delta P\in R^{n}$ of pressure gradients. We obtain *Q* by premultiplying Δ*P* with the diagonal conductance-matrix $G\in R^{n\times n}$ ; this scales the pressure gradient across each edge by the corresponding edge conductance.

Q=−GMP. (3)

In our model, we consider blood as an incompressible fluid and determine the unknown nodal pressures by imposing mass conservation at all nodes. Consequently, the net flow at any given node *i* is zero (Eq. 4). Here, *Q*_*ij*_ is positive when flow enters node *i* $\left(Q_{i\leftarrow j}\right)$ , negative when flow leaves node *i* $\left(Q_{i\to j}\right)$ , and A(i) denotes the set of nodes that are adjacent to *i*.

$$\underset{j\in A\left(i\right)}{\sum }Q_{ij}=0.$$ (4)

Over *m* nodes in the network, the vector *M* ^*T*^ *Q* ( $M^{T}\in R^{m\times n}$ is a divergence operator which is given by the transpose of *M*) defines the flow conservation at all nodes excluding the terminal nodes, where the flow boundary conditions are specified. The non-zero entries of vector $\tilde{q}\in R^{m}$ (Eq. 5) contain the values of inflow or outflow rates at the boundary nodes (Erlich et al., 2019). In addition to the flow rate, we specify one value of known pressure at the inlet *p*_*in*_ or outlet *p*_*out*_. The values of these boundary conditions were specified based on experimental measurements of blood flow velocities reported in Table 1.

$${\tilde{q}}_{i}=\left\{\begin{array}{cc} -Q_{in},\; & \text{if}\;i=\text{inlet node}\\ Q_{out},\; & \text{if}\;i=\text{outlet node}\\ 0,\; & \text{otherwise} \end{array}\right.$$ (5)

$$M^{T}GMP=\tilde{q}.$$ (6)

Here, *M* ^*T*^ *GM* is a square matrix. After substitution of known pressures in the vector P, the columns of *M* ^*T*^ *GM* scaled by the values of known pressure are shifted to the RHS of Eq. 6. This operation results in a non-square matrix on the LHS of Eq. 6. The resulting system of linear equations is solved for the unknown nodal pressures by finding the pseudoinverse (Golub and Pereyra, 1973), which is the generalization of inverse for rank-deficient matrices. Pseudoinverse was computed in MATLAB using singular-value decomposition. From the estimated nodal pressures, the centerline velocity *u*_*ij*_ and the volumetric flow rates are computed using Eq. 2.

### 2.4 Advection–Dispersion of Chemical Species in the Blood Vessel

#### 2.4.1 Domain Discretization

The axial lines (edges) of the pipe network in Figure 3A were spatially discretized to set up the computational domain and study the transport of biochemical species in the microvasculature. For discretizing the edges into 1D elements, in Gmsh (Geuzaine and Remacle, 2009), the vasculature was represented as a geometry with point and line entities. The length of the mesh elements, denoted by *h* in Figure 3B, was constrained by specifying the maximum and minimum characteristic lengths, i.e., *h* ∈ (*c*_*l*_ + *δ*, *c*_*l*_—*δ*). We derive the characteristic length *c*_*l*_ based on the average diameter of a biological cell, approximately 11.5 *μ*m, calculated from the volume ranges reported in the pancreas (Pisania et al., 2010; Parween et al., 2016), and we consider a deviation *δ* of 2.5 *μ*m from *c*_*l*_.

#### 2.4.2 Domain Volume Elements

Each node in the discretized domain forms the center of the volume surrounding it. For instance, the volume of *i*th node $V_{i}^{bv}$ (Equation 7) located at the bifurcation point, depicted in Figure 3B**,** is the sum of half-cylinder volumes formed between nodes i–j, where j ∈A(i) (Reichold et al., 2009). Here, A(i) is the set {*i*-1, *i*+1, *i*+2} of nodes adjacent to *i*, and *A*_*ij*_ and *l*_*ij*_ are the cross-sectional area and length of the cylindrical volume between nodes i–j, respectively. At the junction nodes, we merge the half-cylinder volumes of the adjoining edges and equate the sum to a spherical volume (Figure 3C). Therefore, each branch in the network is dissected into cylindrical elements, and the branches are assembled together by the spherical elements at the junctions. Since the rate of momentum transport is three orders of magnitude greater than the rate of mass transport, the accumulation term at any N-furcation junction is zero while solving for flow field and non-zero while solving the mass transport problem. As an example, for glucose species, the ratio of momentum to mass transport defined by the Schmidt number *ν*/*D* is around 4,000.

$$V_{i}^{bv}=\frac{1}{2}\underset{j\in A\left(i\right)}{\sum }A_{ij}l_{ij}.$$ (7)

#### 2.4.3 Equations

The continuous formulation of the advection–dispersion physics is given by the following partial differential equation, which describes the solute mole balance:

$$A\frac{\partial C^{bv}}{\partial t}=\nabla .\left(-QC^{bv}+DA\nabla C^{bv}\right).$$ (8)

Here, *A* denotes the area of flow, *D* is the dispersion coefficient of species, Q denotes the flow field, and *C* ^*bv*^ is the scalar concentration of a chemical species. To shift from the continuous to discrete counterpart, first we assign the scalar concentration field *C*_0_ to all vertices, and the flow field and dispersion coefficients form the edge weights. The net change in the molar concentration of a species $C_{i}^{bv}$ in the control volume $V_{i}^{bv}$ at the *i*th node of the blood vessel depends on the contributions from advective $J_{i}^{A}$ and dispersive fluxes $J_{i}^{D}$ presented in Equation 9. The *influx* and *outflux* of $J_{i}^{A}$ are the summation of molar fluxes through the edges feeding into *i* (*j* → *i*) and leaving *i* (*i* → *l*), respectively, and the molar flux through an edge is the flow-weighted concentration of the compartment from which the oriented edge originates (Chapman, 2015; Hošek and Volek, 2019). The third term contributes to the dispersive transport. Furthermore, A(i) is the set that contains nodes that are adjacent to node *i*, $A_{+}$ *(i)* is the set of in-neighbor nodes of *i*, $A_{-}$ *(i)* is the set of out-neighbor nodes of *i*, and *D*_*ik*_, *A*_*ik*_, and *l*_*ik*_ are the dispersion coefficient, cross-sectional area, and length of edge *e*_*ik*_, respectively.

$$\begin{array}{c} V_{i}^{bv}\frac{dC_{i}^{bv}}{dt}=\underset{\text{advection flux}\;J_{i}^{A}}{\underset{︸}{\underset{\text{influx}}{\underset{︸}{\underset{j\in A_{+}\left(i\right)}{\sum }C_{j}^{bv}Q_{j\to i}^{in}}}-\underset{\text{outflux}}{\underset{︸}{\underset{l\in A_{-}\left(i\right)}{\sum }C_{i}^{bv}Q_{i\to l}^{out}}}}}\\ \;+\underset{\text{dispersion flux}\;J_{i}^{D}}{\underset{︸}{\underset{k\in A\left(i\right)}{\sum }\underset{D_{ik}^{Q}}{\underset{︸}{\frac{D_{ik}A_{ik}}{l_{ik}}}}\left(C_{k}^{bv}-C_{i}^{bv}\right)}} \end{array}$$ (9)

$$D_{ik}={\tilde{D}}_{ik}+\frac{u_{ik}^{2}r_{ik}^{2}}{48{\tilde{D}}_{ik}}.$$ (10)

The dispersion coefficient is determined from Aris–Taylor’s relation in Eq. 10. Here, ${\tilde{D}}_{ik}$ , *u*_*ik*_, and *r*_*ik*_ are the diffusion coefficient of a species, centerline velocity, and radius of *e*_*ik*_, respectively.

$$V^{bv}\frac{dC^{bv}}{dt}=M^{T}QM^{o}C^{bv}-M^{T}D^{Q}MC^{bv}.$$ (11)

For setting up the discrete advection–diffusion equation on the network, we express the first and second order partial derivatives in Eq. 8 in terms of weighted advection Laplacian proposed by Rak et al. (Rak, 2017) and weighted diffusion Laplacian, respectively. This yields a system of ordinary differential equations shown in Eqs 11 and 12. In Eq. 11, $D^{Q}\in R^{n\times n}$ is the diagonal matrix with diagonal entries the volumetric dispersion coefficient *DA*/*l* of each edge specified in Eq. 9, $Q\in R^{n\times n}$ is the diagonal matrix with diagonal entries the volumetric flow rate of each edge, $M^{o}\in R^{n\times m}$ is the modified incidence matrix (Rak, 2017; Erlich et al., 2019), *M* ^*T*^ *D* ^*Q*^ *M* is the weighted dispersion Laplacian matrix $L^{D}\left(G\right)\in R^{m\times m}$ , and *M* ^*T*^ *QM* ^*o*^ is the weighted advection Laplacian matrix $L^{Q}\left(G\right)\in R^{m\times m}$ (Eq. 12).

$$V^{bv}\frac{dC^{bv}}{dt}=L^{Q}C^{bv}-L^{D}C^{bv}.$$ (12)

### 2.5 Advection–Dispersion-Reaction

#### 2.5.1 Domain

We adapt the Krogh cylinder approach presented in multiscale models (Thurber and Weissleder, 2011; Chalhoub et al., 2007; Berndt et al., 2018b) and approximate the layer of tissue surrounding the vessels as a hollow cylindrical volume element *ω* ^*t*^ illustrated in Figure 3D. The outer diameter of the region *ω* ^*t*^ is given by the summation of *d*_*bv*_ and *d*_*t*_. A value of 12.4 µm was used in our model for *d*_*t*_. This value is derived from the physical volume of an insulin-secreting beta cell (1020* μm* ^*3*^) (Finegood et al., 1995) located in the islets of pancreas.

#### 2.5.2 Equations

$$\begin{array}{cc} V_{i}^{bv}\frac{dC_{i}^{bv}}{dt} & =\underset{\text{advection flux}\;J_{i}^{A}}{\underset{︸}{\underset{j\in A_{+}\left(i\right)}{\sum }C_{j}^{bv}Q_{ji}^{in}-\underset{l\in A_{-}\left(i\right)}{\sum }C_{i}^{bv}Q_{il}^{out}}}\\ & +\underset{\text{dispersion flux}\;J_{i}^{D}}{\underset{︸}{\underset{k\in A\left(i\right)}{\sum }\frac{D_{ik}A_{ik}}{l_{ik}}\left(C_{k}^{bv}-C_{i}^{bv}\right)}}\\ & -\underset{\text{exchange flux}\;J_{i}^{E}}{\underset{︸}{V_{i}^{cell}j_{i}^{E}}}. \end{array}$$ (13)

In the last term of Eq. 13, $j_{i}^{E}$ (Eq. 15) is the net exchange rate that governs the bidirectional transport between tissue compartment $\omega_{i}^{t}$ and blood vessel compartment $\Omega_{i}^{bv}$ of the functional unit. The uptake or release flux $J_{i}^{E}$ is computed by multiplying $j_{i}^{E}$ with $V_{i}^{cell}$ since the maximal rates are often reported in per unit volume of a biological cell. This ensures mole balance when a species moves from $\omega_{i}^{t}$ to $\Omega_{i}^{bv}$ which differs in volume. Furthermore, $C_{i}^{cell}$ is the concentration of species in the tissue cell that interacts with *i*th node of Ω^*bv*^, the half-saturation constant *K*_*m*_ quantifies the affinity of a transporter protein or an enzyme for a metabolite, and *V*_*m*_ is the maximal rate of metabolite transport. When $\Omega_{i}^{bv}$ is encompassed by $\omega_{i}^{t}$ , interaction between the node associated with both the compartments exists, and $J_{i}^{E}$ appears as a source or sink term in Eq. 13. At the junction nodes of the blood vessel where no interaction exists, $J_{i}^{E}$ is zero.

$$V^{bv}\frac{dC^{bv}}{dt}=M^{T}QM^{o}C^{bv}-M^{T}D^{Q}MC^{bv}-J^{E}$$ (14)

$$j_{i}^{E}=\frac{V_{m}}{K_{m}}\left(\frac{C_{i}^{bv}-C_{i}^{cell}}{1+\frac{C_{i}^{cell}}{K_{m}}+\frac{C_{i}^{bv}}{K_{m}}}\right).$$ (15)

As an example, we consider a minimal model of glucose metabolism in *β*-cells in the islets. Under basal conditions, the concentration of glucose and lactate are at the basal level in the bloodstream. In the fed state, glucose transporters sense the high blood glucose level and export glucose from Ω^*bv*^ to Ω^*t*^. The glucose-to-lactate conversion in Ω^*t*^ is presented as a lumped reaction in our model for simplicity. Ω^*t*^ acts as a source of lactate, and the lactate transporter facilitates its release into the bloodstream. The direction of the exchange flux is dictated by the concentration gradient *C* ^*bv*^—*C* ^*cell*^ across the vessel wall.

### 2.6 Cell

The mole balance of each species in the cell domain *ω* ^*t*^ is modeled by

$$V^{cell}\frac{dC_{i}^{cell}}{dt}=Nv,$$ (16)

where N is the stoichiometric matrix, and *v* is the reaction flux vector. The rate expressions of the kinetic reactions and metabolite transporters and the values of the kinetic parameters used in the model are presented in Table 2. The values of half-saturation constants, *K*_*m*_ and *V*_*m*_, were chosen from *panmin* (König and Deepa Maheshvare, 2021), a minimal model of glucose metabolism and insulin secretion in the pancreatic *β*-cell. The *V*_*m*_ values from the minimal model were scaled in this study so that the reaction fluxes are comparable to the diffusive and convective fluxes. To apply our framework for predictive modeling in clinical applications, the *V*_*m*_ values can be determined by model calibration to achieve good agreement between model predictions and experimental measurements. Steady-state and transient values of the metabolite and flux distributions measured from biochemical assays can be used as the inputs for calibrating the tunable parameters in the model (Berndt et al., 2018b). Since the focus of this study is in introducing and illustrating the applicability of our mathematical framework for bridging multiple scales, the parameters were not calibrated in the glucose–lactate test system presented here.

**TABLE 2:** Reactions, rate laws, and parameter values of exchange and cellular reactions.

Flux	Reaction	Rate law	Parameter value (*V*_*m*_ in M/min, *K*_*m*_ in mM)
*v*_*TI*_	*A* ^*bv*^ *⇌ A* ^*cell*^	$\frac{V_{m}^{T1}\left(A^{bv}-A^{cell}\right)}{K_{m,A}^{T1}+A^{bv}+A^{cell}}$	$V_{m}^{T1}$ = 10, $K_{m,A}^{T1}$ = 1.0
*v*_*E*_	*A* ^*cell*^ → 2*B* ^*cell*^	$\frac{V_{m}^{E}A^{cell}}{A^{cell}+K_{m,A}^{E}}$	$V_{m}^{E}$ = 0.01, $K_{m,A}^{E}$ = 4.5
*v*_*T*2_	*B* ^*cell*^ *⇌ B* ^*bv*^	$\frac{V_{m}^{T2}\left(B^{cell}-B^{bv}\right)}{K_{m,B}^{T2}+B^{cell}+B^{bv}}$	$V_{m}^{T2}$ = 10, $K_{m,B}^{T2}$ = 0.5

A: glucose, B: lactate, T1: glucose transporter *glcim*, T2: lactate transporter *lacex*, and E: glucose to lactate converter *glc2lac* are the tags used in the mathematical model.

## 3 Results and Discussion

The discrete model framework developed allows bridging the cell-to-vessel exchange and explicitly modeling the cellular dynamics. In this section, the mathematical formulations presented in Section 2 are verified using several test systems, and the results are compared with finite element analysis in COMSOL. The analysis includes solving two steps that are coupled: first, a flow field analysis which involves computation of pressure and velocity distribution in the blood vessel branches; and second, analysis of the spatio-temporal evolution of the concentration fields.

We first present the results of the pressure gradient and flow distribution observed across the islet vasculature. After validating the results of nodal pressures and edge velocities with the results from COMSOL, we proceed with the simulations of advection–dispersion dynamics of glucose species in the blood vessel. Here, we compare the transient change in the blood glucose concentration obtained from our discrete model versus COMSOL simulations for islet and mesentery networks. Next, we investigate the effect of change in the perfusion pattern on glucose distribution by varying the inlet and outlet locations in the tumor vasculature. Furthermore, we examine the effect of different pressure gradients applied across the network and the effect of glucose doses supplied at the inlet on metabolite rise times observed in the islet vasculature.

All the 3D visualizations of flow and concentration fields presented in this article are rendered using *vedo* (Musy et al., 2021), a python-based module for analyzing and visualizing multidimensional point-cloud, mesh, and volume data.

### 3.1 Comparison of Flow and Concentration Fields

To illustrate how the results from our discrete formulation compare with the finite element implementation available in COMSOL, we first solve the static flow problem (Eq. 6) and use the flow profile for simulating the advection–dispersion dynamics (Eq. 12) in the islet vasculature.

**FIGURE 4:**
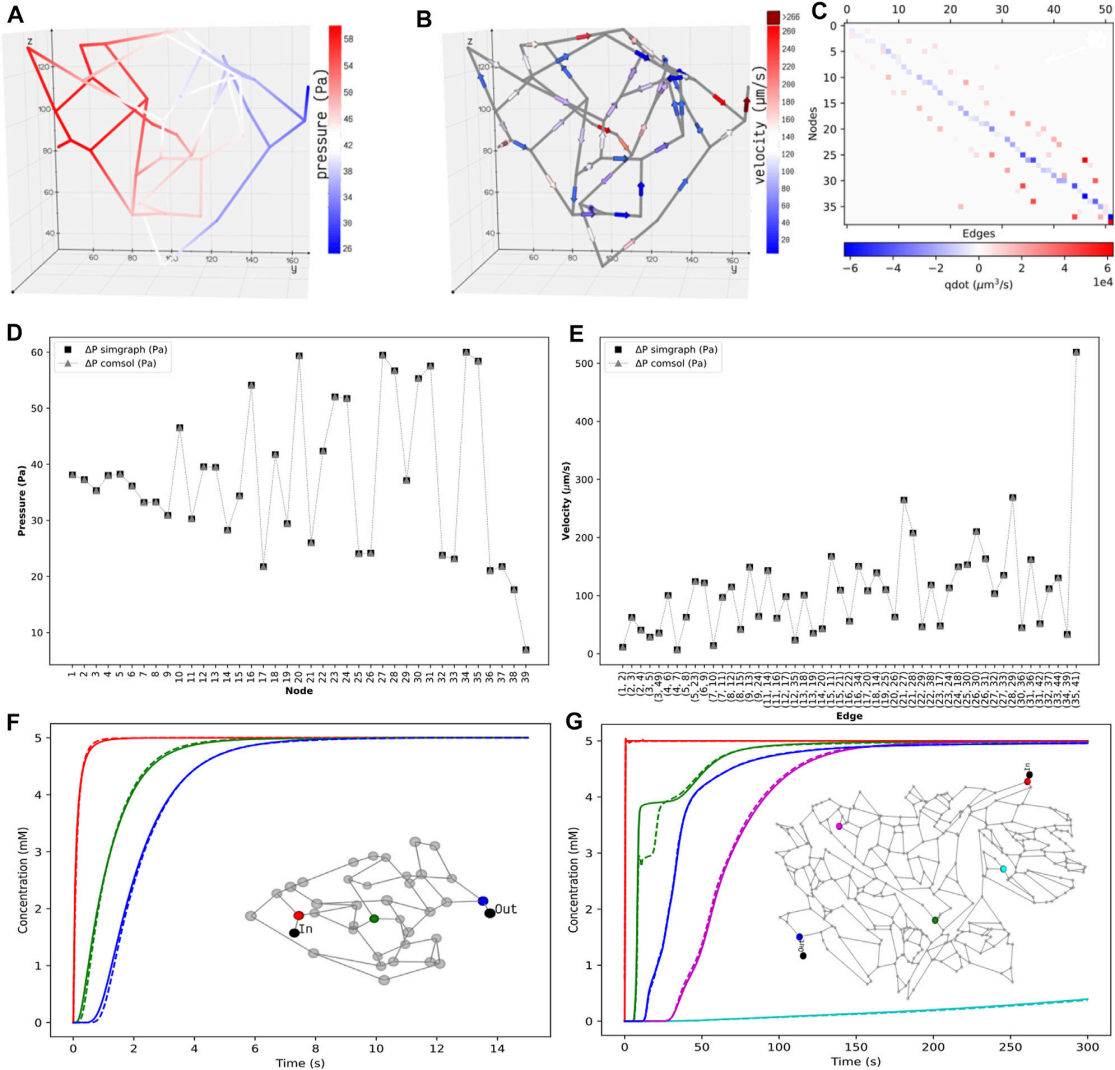
Distribution of the static properties of islet vasculature. **(A)** Pressure gradient from inlet to outlet and **(B)** directional flow from inlet to outlet. **(C)** Flow conservation at all nodes. The summation of flows through the edges entering and leaving each node, except the boundary nodes, adds to zero. Validation of static results of the islet vasculature. **(D)**: Comparison of nodal pressures between the current work and COMSOL simulation. **(E)**: Comparison of edge velocities between the current work and COMSOL simulation. Comparison of the concentration profiles at various locations in the islet and mesentery vasculatures. **(F)** Scalar concentrations observed at positions 12.07 *μ*m (red), 54.85 *μ*m (green), and 110.27 *μ*m (blue) from the inlet node. **(G)** Scalar concentrations observed at positions 1,267.78 *μ*m (blue), 1,692.3 *μ*m (cyan), 3,476.96 *μ*m (green), 5,085.33 *μ*m (magenta), and 6,319.57 *μ*m (blue) from the inlet node. Solid and dashed lines indicate the transient change in glucose concentration from our model and COMSOL, respectively.

The pressure and velocity fields computed across the islet vasculature are shown in Figures 4A,B. We observe a net pressure drop of 34.83 Pa for an inlet pressure and flow rate of 60 Pa and 3.76 nl/min, respectively. The velocity distribution lies in the range reported by Diez et al. (Diez et al., 2017). The conservation of flow at each node is cross-verified by computing the divergence of the flow field *M* ^*T*^ *Q*, and the visualization is presented in Figure 4C.

#### 3.1.1 Validation in COMSOL Multiphysics

Here, we describe the simulation implementation of the coupled multiphysics problem in COMSOL to validate the flow fields and concentration fields of advection–dispersion simulations from our discrete model. *Geometry:* The generation of the geometrical model of the islet and mesentery vasculature (Figure 2A,D) was automated using an AutoLISP script (Mac, 2020). The coordinates of points and the connectivity information of the lines were specified as inputs for creating the CAD geometry. The DXF file containing the geometry data was imported into COMSOL, and the *normal* mesh size was used to generate the mesh elements. *Parameters:* Values of the diffusion coefficient of glucose species, viscosity of blood, inlet pressure and outlet flow rate specified in Table 1, and diameter of all branches in the vasculature were defined as input parameters. *Computation*: Fluid flow was studied as a static problem in the *Pipe Flow* Module considering blood as a Newtonian fluid. This stationary problem was solved as a linear system using the *direct* solver by specifying the pressure and flow boundary conditions. Then, one-way coupling of the flow physics was established with the *Transport of Dilute Species in Pipes* module to solve for the time-dependent advection–dispersion physics of glucose species in the finite element solver. For this transient simulation, a value of 5 mM was used for the Dirichlet boundary condition defined at the inlet, and the mass outflow was modeled by setting the diffusive flux to zero. The initial concentration was set to zero in the volume elements of our discrete framework. In COMSOL, the concentration was initialized to zero, and a smoothed step function was applied to avoid discontinuity with the boundary condition.

Figures 4D,E show that the nodal pressures and edge velocities computed from our model are consistent with the results from COMSOL for the islet vasculature. The time-varying concentration profile of glucose species obtained from our discrete model is compared with COMSOL simulations at nodes highlighted in the inset of Figure 4F. After comparing the results of the islet vasculature containing 52 edges and 125 discretized elements in our discrete model, we further extend this analysis to compare the results (Figure 4G) of the large mesentery network with 489 edges and 9,033 discretized elements. Supplementary videos S1 and S2 show the comparison of evolution of concentration profiles from our model versus the results from COMSOL for the entire region of the islet and mesentery vasculature, respectively.

#### 3.1.2 Scalability

The procedure involved in solving the partial differential equations can be split into two steps: assembly step and solve step. In the assembly step, discretization is performed, and matrices are generated for the second step which solves the system. For the islet vasculature, we report the time taken for solving the stationary fluid flow and the transient advection–dispersion dynamics for a time span of 15 s. Our discrete method implementation in MATLAB takes 7 s, and the finite element solver in COMSOL takes 10 s. For the mesentery network, running the advection–dispersion dynamics for a time span of 300 s takes 267 and 168 s in MATLAB and COMSOL implementations, respectively. We provide the sparse Jacobian pattern as an additional input to *ode15* *s* to speed up the compute time. Solving the same system in Julia using the QNDF method, which is a translation of MATLAB’s ode15 s, gives a speed up of 127x when compared to that of COMSOL. A relative error tolerance of 1e-3 is set for carrying out all simulations, and an absolute error tolerance of 1e-6 is set for both ode15 s and QNDF.

Some of the challenges involved in expanding this approach to a vascular network composed of arterioles, arteries, and veins would be in scaling flow parameters and resistances to flow (such as molecular interaction in capillary versus viscosity in larger vessels) to fit the formulation so that the model still retains its physical fidelity. Computationally, there can be stiffness in the differential equations and condition numbers of matrices may be affected, especially when both capillary and larger vessels are present in the model. This may need smaller time steps in simulation and regularization of affected matrices.

### 3.2 Influence of Flow Topology on Scalar Transport

Engineered perfusable vasculatures have been useful for investigating the structure–function relationship of complex vasculatures (Kinstlinger et al., 2020). Computational models that capture the influence of flow topology on the scalar transport will enable experimental scientists to design and test the efficacy of optimized drug delivery systems.

**FIGURE 5:**
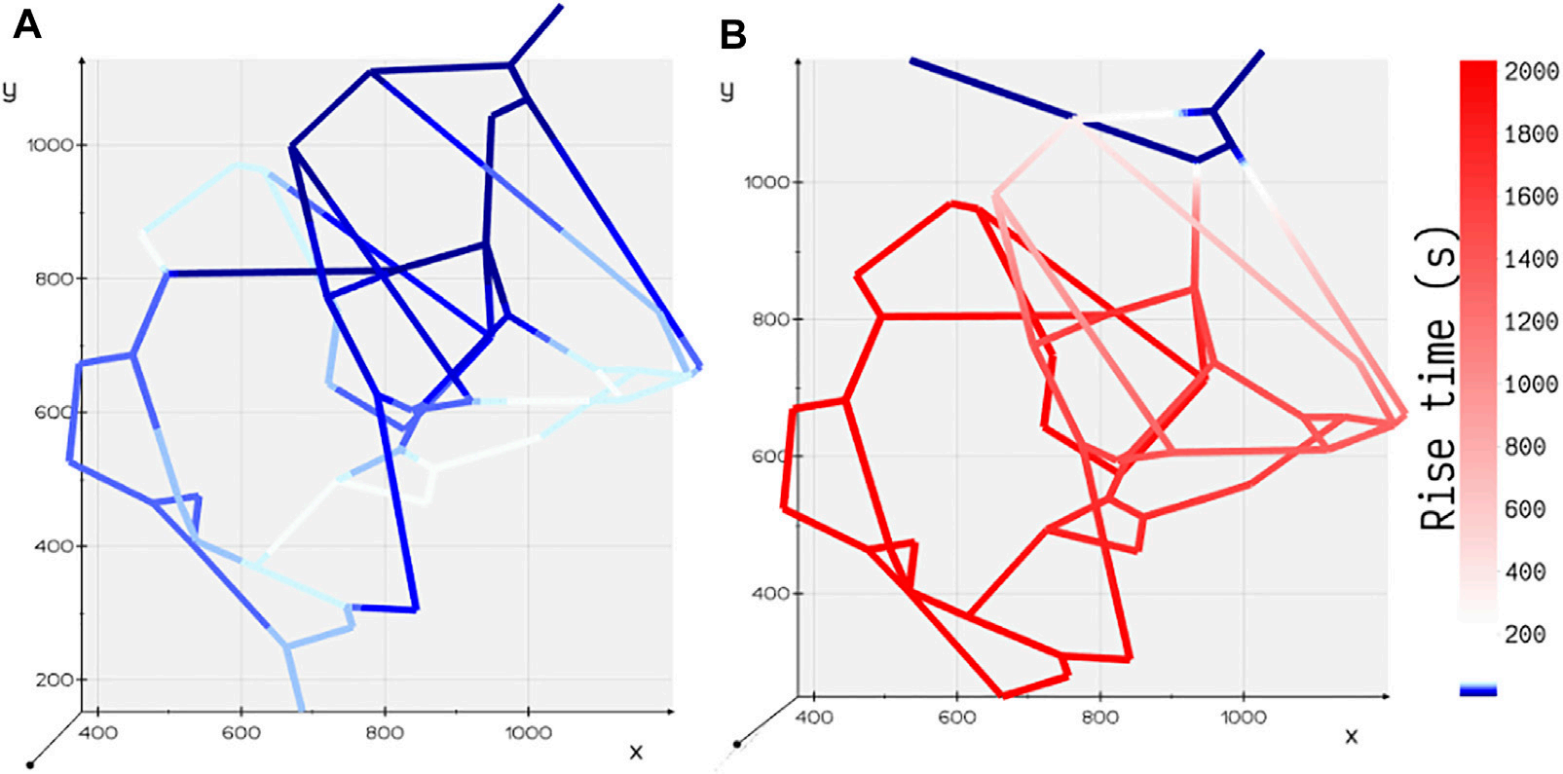
Comparison of rise time *t*_*r*_ for two different configurations of tumor vasculature. **(A)** Tumor design 2: the outlet is positioned away from the inlet, and the rise time is shorter in this configuration. **(B)** Tumor design 1: the outlet is positioned closer to the inlet, and the rise time is longer in this configuration.

Motivated by the experiments carried out by Chen et al. (Chen et al., 2020) on microfluidic devices imprinted with blood vessel vasculatures, we study the effect of variation in perfusion patterns on the metabolite rise times *t*_*r*_ in two different geometric configurations (Figures 2B,C) of the tumor vasculature examined in their study. Flow distribution was computed for an inlet pressure of 60 Pa and fluid flow rate of 23.8 nl/min. Spatial distribution of glucose species was obtained, and the rise time in each volume element $\Omega_{i}^{bv}$ is computed by finding the time taken for the concentration to rise from 10 to 90% of the steady-state value. Figure 5 illustrates the time taken for the distribution of glucose in *tumor design 1* (Figure 5B) is much greater than that in *tumor design 2* (Figure 5A). These findings support the dye distribution patterns observed in these two configurations reported in Chen et al. Supplementary Video S3 shows the distribution of glucose computed by our discrete model for both the configurations of tumor vasculature.

### 3.3 Functional Coupling of Blood Vessel–Cell Exchange

In the mathematical framework presented in Section 2, it is practical to model heterogenous cell types exhibiting heterogenous enzyme activity at different vascular and cell densities observed under various pathophysiological conditions.

**FIGURE 6:**
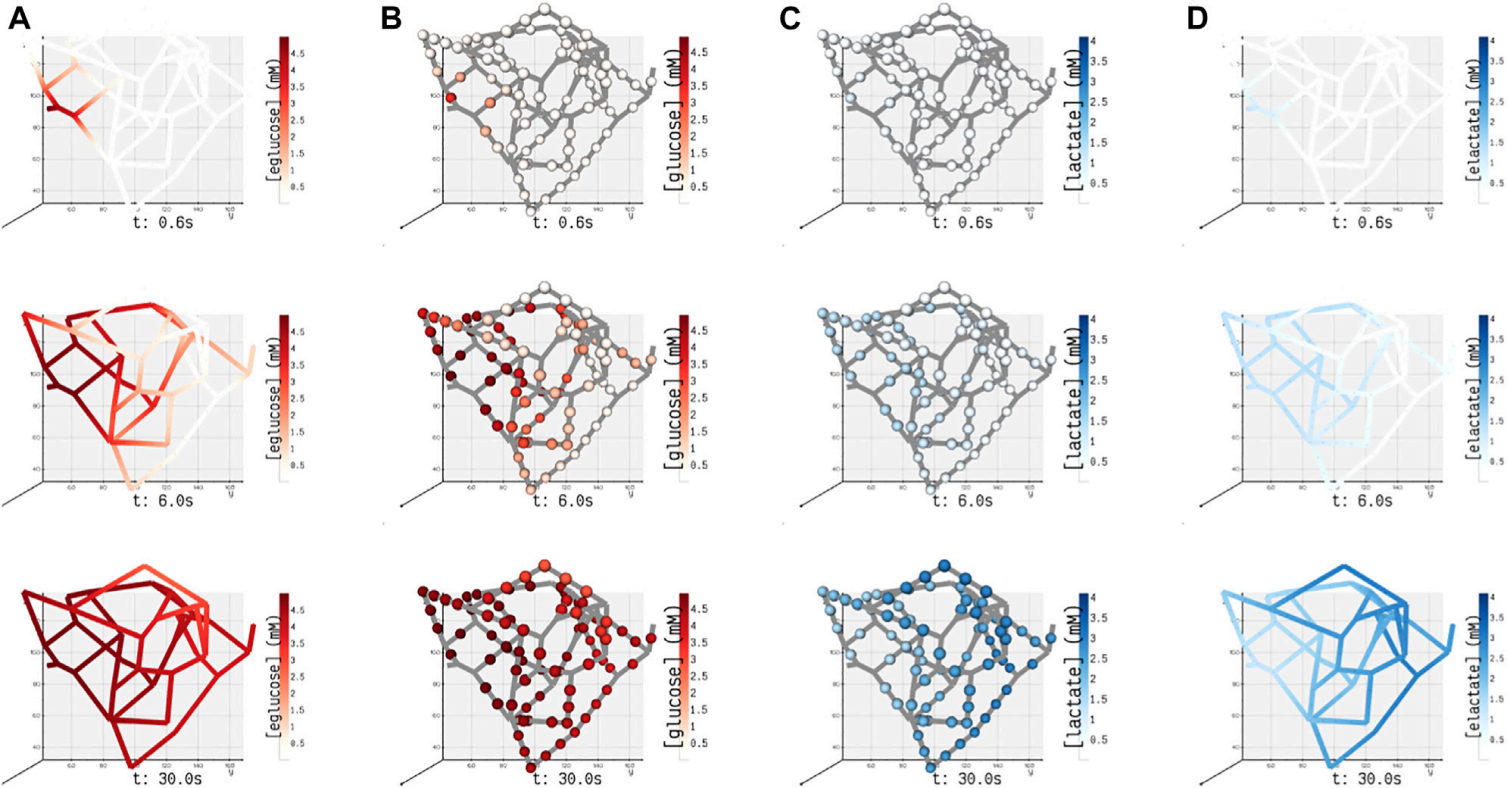
Spatial concentrations of metabolites in the blood vessel and tissue domains of the islet vasculature observed at three different instants in time (0.6, 6, and 30 s). **(A)** (eglucose)/ **(B)** (elactate) and **(C)** (glucose)/ **(D)** (lactate) denote the concentration of glucose/lactate species observed in blood vessel Ω^*bv*^ and tissue *ω* ^*t*^ compartments, respectively. In panel **(A)** and **(D)**, the concentration of cylindrical and spherical volume elements embedded in the blood vessel domain is simulated, and the gradient displayed along the length of the blood vessel is generated by interpolation. In panels **(B)** and **(C)**, the color-coded spheres represent the concentration evaluated in the annular region of *ω* ^*t*^.

In this study, for the ease of demonstration of our method, we present the results of a minimal model of cellular glucose metabolism with uniform enzyme activity and homogenous cell type exchanging biochemicals with bloodstream. In Figure 6, we present the volumetric spatial analysis of the islet vasculature enveloped by a layer of homogenous cell mass, which forms the annular region of the tissue domain *ω* ^*t*^. The extracellular glucose and lactate concentrations in Ω^*bv*^ are initialized to 5 and 1.2 mM at the network inlet, respectively. The intracellular concentrations of glucose and lactate in *ω* ^*t*^ are initialized to zero. Due to the high concentration of glucose in the bloodstream, the gradient established between Ω^*bv*^ and *ω* ^*t*^ in response to the advective–dispersive transport promotes the uptake of glucose by the annular region *ω* ^*t*^. The cellular enzymes metabolize glucose-to-lactate, and the concentration of lactate in *ω* ^*t*^ rises above the basal value in the bloodstream. This gradient drives the export of lactate into the bloodstream until the system equilibrates.

It is known from clinical observations that vascular phenotypes alter in cohorts with disease conditions such as diabetes and tumor. For example, in a tumor condition, the glycolytic enzymes are upregulated to produce more energy molecules that aid in the rapid proliferation of tumor cells (Rojas et al., 2018; St Clair et al., 2018). As a result, glucose–lactate dynamics is perturbed when compared to normal cells. In case of diabetes, histopathological and image reconstruction studies reveal that a decrease in the cell mass and reduction in vascular density can alter insulin release patterns (Richards et al., 2010; Cohrs et al., 2017). The framework proposed here can be useful in such clinical applications for carrying out systematic analysis in which 1) the change in vascular density can be induced in the form of mutations in the network (e.g., by deleting or inserting blood vessels) to examine the effect of anatomical changes on functional response of a tissue; 2) the alterations in the expression levels of enzymes quantified as fold changes in proteomics studies (Haythorne et al., 2019; Malinowski et al., 2020) can be mapped to the reaction velocities (i.e., Vm, a function of enzyme abundance, can be scaled using fold change) to examine the effect of genetic or environmental perturbations on cellular dynamics.

### 3.4 Sensitivity of Concentration Dynamics to ΔP and Glucose Dose

**FIGURE 7:**
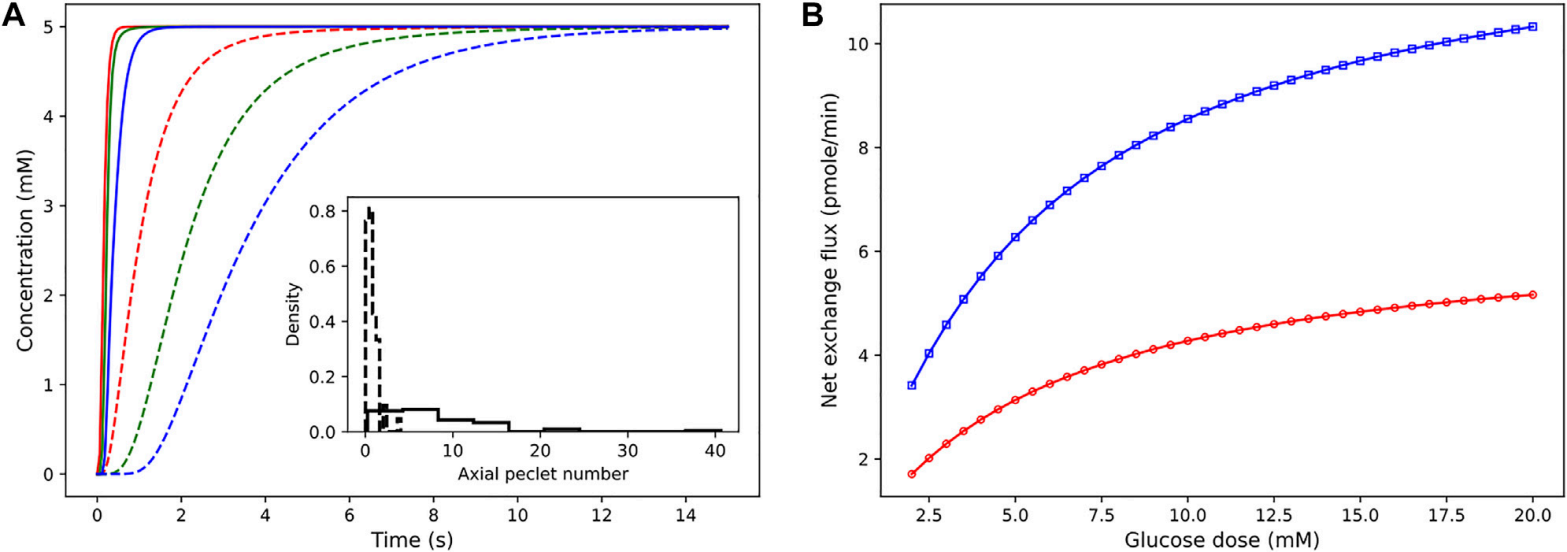
Sensitivity of concentration dynamics to ΔP and glucose dose. **(A)** Left: influence of the varying pressure gradient across the network on metabolite rise times. Glucose concentration observed at positions 12.07 *μ*m (red), 54.85 *μ*m (green), and 110.27 *μ*m (blue) from the inlet node. Inset displays the distribution of the Peclet number obtained by computing *ul/* $\tilde{D}$ for each branch. Dashed and solid lines indicate the dynamic change in concentration observed at 20Pa and 200Pa, respectively. **(B)** Right: variation in the net glucose uptake (red) and net lactate release (blue) from the cells in response to change in glucose dose is displayed.

The pressure conditions observed in a vascular tissue may vary due to several physiological factors. To study the influence of the pressure gradient on the time taken to reach the steady-state concentration, we vary ΔP across the vasculature by specifying the inlet pressure and zero outlet pressure. Figure 7A illustrates the concentration profiles generated by simulating the advection–dispersion dynamics of glucose species by varying the pressure drop from 20 Pa to 200 Pa. When ΔP is high, the velocity of fluid is high in each branch. Consequently, convective flow dominates over dispersion, and this results in short rise times. The effect of change in the pressure gradient results in change in transit time of the fluid from 90.76 s at ΔP = 20 Pa to 9.08 s at ΔP = 200Pa. In Figure 7B, we show the sensitivity of the net glucose uptake flux and net lactate release flux to different glucose doses set at the inlet. It is observed that with an increase in the glucose dose the tissue units uptake more glucose from blood, and this drives the formation of lactate in the tissue subdomains. The excess lactate is then transported to the vessel subdomains until equilibrium is attained and the driving potential is zero.

In conclusion, here we have presented a mathematical framework for understanding the multiscale connectivity existing in the functional networks of tissues. The test cases presented previously demonstrate how experimental data from different sources (e.g., kinetic data available in databases such as SABIO-RK (Wittig et al., 2012) and BRENDA (Chang et al., 2021), proteomics and metabolomics data, imaging data of vascular phenotypes, and flux measurements from perfusion experiments) can be encoded in our framework to build explicit models of cellular and vessel-to-cell interaction dynamics to better predict pathomechanisms. At the intra-organ scale, this work can be further extended to include cell-to-cell communication networks and decipher the order of communication occurring in the microenvironments with different vasculature architectures (e.g., periphery to the center, center to periphery, and one pole to other patterns in islets (El-Gohary and Gittes, 2018)) and cytoarchitectures (e.g., mantle-core and heterogenous distribution of *β* and *α* cells in islets (Dolenšek et al., 2015)). The metamodel is easily mutable; it is possible to induce different vascular phenotypes and disease states by altering vascular density and incorporating fold changes of metabolic and enzyme concentrations in the cellular units. To further research efforts involved in carrying out virtual experiments of inter-organ communication, our framework can be utilized for piecing together the top-down and bottom-up modeling approaches and accommodates each organ at the desired spatial resolution. For example, a comprehensive understanding of multi-organ disease states such as diabetes can be developed by interpreting the intra- and inter-organ interaction as communication occurring within “network of networks”; in whole-body models, the inter-organ communication can be modeled by abstracting organs as compartments forming nodes of the global network, and the detailed local dynamics occurring in the functional networks of an organ can be modeled by including subnetworks in the global network.

## Data Availability

The original contributions presented in the study are included in the article/Supplementary Material; further inquiries can be directed to the corresponding author.
