# Supplementary material for: A Graph-Based Framework for Multiscale Modeling of Physiological Transport
Source: Front Netw Physiol. 2022 Jan 12;1:802881. doi: 10.3389/fnetp.2021.802881 (PMC10013063; doi:10.3389/fnetp.2021.802881)
Supplement: Supplementary file 1 [file DataSheet2.PDF]

islet.mph
mesentry.mph

Please download the files from the PDF attachment for testing.
