# Supplementary figures and images for: A Graph-Based Framework for Multiscale Modeling of Physiological Transport
Source: Front Netw Physiol. 2022 Jan 12;1:802881. doi: 10.3389/fnetp.2021.802881 (PMC10013063; doi:10.3389/fnetp.2021.802881)

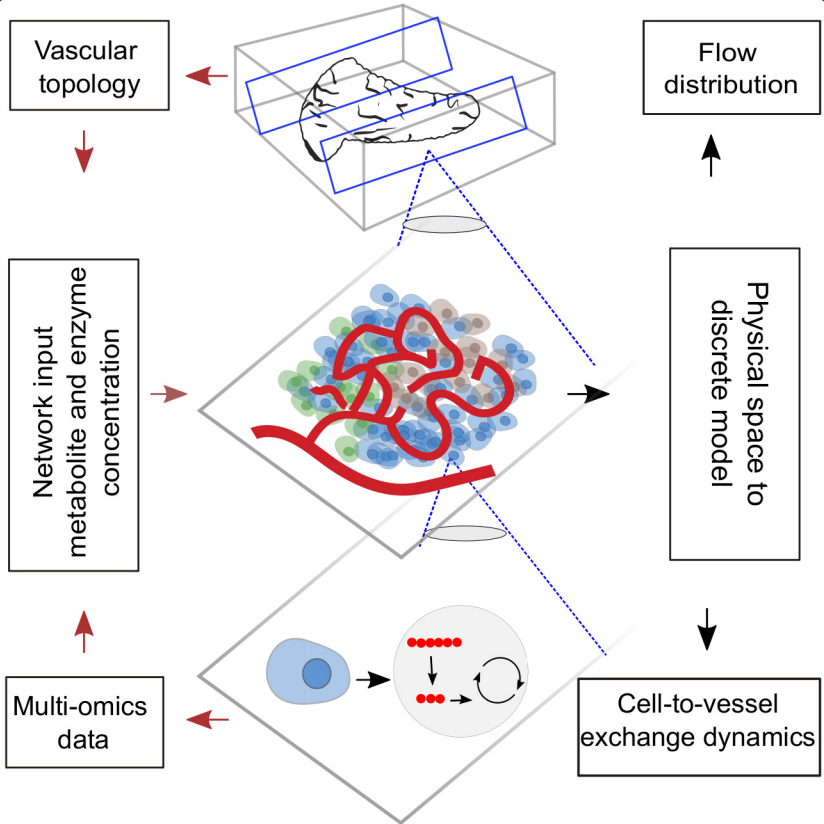

Supplement: Supplementary file 3 [file DataSheet3.PDF]
