# Supplementary material for: A Graph-Based Framework for Multiscale Modeling of Physiological Transport
Source: Front Netw Physiol. 2022 Jan 12;1:802881. doi: 10.3389/fnetp.2021.802881 (PMC10013063; doi:10.3389/fnetp.2021.802881)
Supplement: Supplementary file 4 [file DataSheet1.PDF]

TABLE I. Notation

$\mathcal{G}$		Weighted graph
m		Number of vertices or nodes in $\mathcal{G}$
n		Number of edges in $\mathcal{G}$
$\mathbb{V}$	$\in \mathbb{R}^m$	Dimension of vertex
$\mathbb{E}$	$\in \mathbb{R}^n$	Dimension of edge
$\mathbb{W}$	$\in \mathbb{R}^n$	Dimension of edge weights
$\mathbb{W}_G$	$\in \mathbb{R}^n$	Edge weight vector with entries the conductance of each edge
$\mathbb{W}_Q$	$\in \mathbb{R}^n$	Edge weight vector with entries the volumertic flow rate along each edge
$\mathbb{W}_{DQ}$	$\in \mathbb{R}^n$	Edge weight vector with entries the volumertic dispersion coefficient of each edge
G	$\in \mathbb{R}^{n\times n}$	Diagonal matrix with diagonal entries $\mathbb{W}_G$
Q	$\in \mathbb{R}^{n\times n}$	Diagonal matrix with diagonal entries $\mathbb{W}_Q$
D^Q	$\in \mathbb{R}^{n\times n}$	Diagonal matrix with diagonal entries $\mathbb{W}_{DQ}$
$M\left(\mathcal{G}\right)$	$\in \mathbb{R}^{n\times m}$	Incidence matrix of $\mathcal{G}$
$M^T\left(\mathcal{G}\right)$	$\in \mathbb{R}^{m\times n}$	Transpose of incidence matrix of $\mathcal{G}$
$M^o\left(\mathcal{G}\right)$	$\in \mathbb{R}^{n\times m}$	Modified incidence matrix of $\mathcal{G}$
$L^D\left(\mathcal{G}\right)$	$\in \mathbb{R}^{m\times m}$	Weighted diffusion laplacian matrix of $\mathcal{G}$ computed as $M^T D^Q M$
$L^Q\left(\mathcal{G}\right)$	$\in \mathbb{R}^{m\times m}$	Weighted advection laplacian matrix of $\mathcal{G}$ computed as $M^T Q M^o$
$\mathcal{A}\left(i\right)$		Adjacent nodes of i
P	$\in \mathbb{R}^m$	Dimension of vector containing nodal pressures
$\tilde{q}$	$\in \mathbb{R}^m$	Dimension of vector containing boundary data
C	$\in \mathbb{R}^m$	Dimension of concentration vector
J	$\in \mathbb{R}^m$	Dimension of exchange-flux vector
V	$\in \mathbb{R}^m$	Dimension of vector containing the volume of nodes

TABLE II. Values of parameters used in transport equation

Symbol	Description	Value/expression	Units	Reference
r_{bv}	Radius of blood vessel		μm	
r_{cell}	Radius of cell		μm	
d_{cell}	Diameter of cell	$2r_{cell}$	μm	
Δx	Length of blood vessel element; distance between two consecutive nodes	d_{cell}	μm	
$r_{bv,in}$	Radius at the inlet of blood vessel		μm	
$u_{bv,in}$	Velocity at the inlet of blood vessel		$\mu m/s$	
A_{cell}	Cross-sectional area of tissue element	$\pi\left((r_{bv}+d_{cell})^2-r_{bv}^2\right)$	μm^2	
A_{bv}	Cross-sectional area of blood vessel element	πR^2	μm^2	
V_{bv}	Volume of blood vessel element	$A_{bv}\Delta x$	μm^3	
V_{cell}	Volume of tissue element	$A_{cell}\Delta x$	μm^3	
P_{in}	Inlet blood pressure		mmHg	
Q_{in}	Volumetric flow rate at the inlet of blood vessel	$u_{bv,in}\pi r_{bv,in}^2$	$\mu m^3/s$	
$\tilde{D}_A$	Diffusion coefficient of species A in blood	5.46E-4	cm^2/min	Berndt et al. (2018)
$\tilde{D}_B$	Diffusion coefficient of species B in blood	7.71E-4	cm^2/min	Berndt et al. (2018)
D_A	Dispersion coefficient of species A in blood		cm^2/min	
D_B	Dispersion coefficient of species B in blood		cm^2/min	
D_A^Q	Volumetric dispersion coefficient of species A in blood	$\frac{D_A A_{bv}}{\Delta x}$	cm^3/min	
D_B^Q	Volumetric dispersion coefficient of species B in blood	$\frac{D_B A_{bv}}{\Delta x}$	cm^3/min	
ν	Viscosity of blood	0.004	Pa.s	Berndt et al. (2018)
J_i^E	Exchange flux		mole/min	

Video descriptions

Supplementary Video S1
Comparison of the evolution of concentration profiles of glucose species, governed by the advection-dispersion physics, computed from our discrete model versus the results from COMSOL simulation for the entire region of the islet vasculature.

Supplementary Video S2
Comparison of the evolution of concentration profiles of glucose species, governed by the advection-dispersion physics, computed from our discrete model versus the results from COMSOL simulation for the entire region of the mesentery vasculature.

Supplementary Video S3
Comparison of the distribution of glucose concentration, governed by the advection-dispersion physics, computed from our discrete model for the design 1 and 2 configurations of the tumor vasculature.

COMSOL file descriptions

Islet Model
COMSOL simulation of the advection-dispersion dynamics of glucose species in the islet vasculature.

Mesentery Model
COMSOL simulation of the advection-dispersion dynamics of glucose species in the mesentery vasculature.

References

Nikolaus Berndt, Marius Stefan Horger, Sascha Bulik, and Hermann-Georg Holzhütter. A multiscale modelling approach to assess the impact of metabolic zonation and microperfusion on the hepatic carbohydrate metabolism. *PLoS computational biology*, 14(2):e1006005, 2018.
